# Outcome in patients with HIV-associated Hodgkin lymphoma treated with chemotherapy using Doxorubicin, Bleomycin, Vinblastine, and Dacarbazine in the combination antiretroviral therapy (cART) era: results of a multicenter study from China

**DOI:** 10.1186/s13027-024-00571-w

**Published:** 2024-04-15

**Authors:** Lirong Xiao, Chaoyu Wang, Sai Ma, Yifan Wang, Liping Guan, Juyi Wu, Wei Zhang, Yao Liu, Yan Wu

**Affiliations:** 1 Department of Medical Oncology, Henan Infectious Disease Hospital, The Sixth People’s Hospital of Zhengzhou, 450000 Zhengzhou, China; 2 Department of Hematology-Oncology, Chongqing Key Laboratory of Translational Research for Cancer Metastasis and Inaffiliationidualized Treatment, Chongqing University Cancer Hospital, 400030 Chongqing, China; 3 Department of Hematology, Peking Union Medical College Hospital, Chinese Academy of Medical Sciences (CAMS) & Peking Union Medical College, 100000 Beijing, China

**Keywords:** HIV, Hodgkin lymphoma, cART, Treatment, Prognosis

## Abstract

Little is known about the outcome for HIV-associated Hodgkin lymphoma (HIV-HL) as this is less common than HIV-negative lymphoma. Therefore, we performed a multi-center study to analyze the clinical characteristics and outcomes of HIV-HL patients in China. Nineteen cases of HIV-HL were diagnosed and treated at three center and including the sixth people’s hospital of Zhengzhou, Peking union medical college hospital, and Chongqing university cancer hospital, between December 2013 and June 2022. Data on the clinical features, laboratory results, response, and prognosis were collected and analyzed. The median age at diagnosis was 43(22–74) years. All patients were infected with HIV through sexual transmission, with ten cases transmitted through man having sex with man (MSM) and nine cases transmitted through heterosexual transmission. Seven patients were diagnosed with lymphoma and found to be infected with HIV. Four cases were in stage III, and fifteen cases were in stage IV. After a median follow up of 46.8(4.0-112.9) months, 17 cases were alive after ABVD regimen chemotherapy combined with combination antiretroviral therapy (cART). The 5-year progression-free survival (PFS) and overall survival (OS) rate were 83.9% and 89.5%,respectively. HIV-HL exhibits an invasive process in clinical practice, and cART combined with ABVD regimen chemotherapy can achieve long-term survival for patients.

## Introduction

HIV-associated Hodgkin Lymphoma (HIV-HL) is a rare subtype of lymphoma, often diagnosed in advanced stages with accompanying B-symptoms. However, with the widespread use of the combination antiretroviral therapy (cART), the survival of HIV-HL patients closely approaches that of HIV-negative Hodgkin lymphoma patients [1]. Existing literature primarily discusses its clinical and pathological characteristics in the form of case reports [2–4]. Currently, there is a lack of large-sample studies. In this study, we conducted a retrospective analysis of clinical data from19 newly diagnosed HIV-HL patients to explore their clinical features, pathological characteristics, and survival outcomes.

## Methods

### Patients and clinical data analysis

We conducted a retrospective analysis of clinical data for newly diagnosed HIV-HL patients aged > 18 years, with a confirmed pathological diagnoses, treated at three medical centers in China, which includes the sixth people’s hospital of Zhengzhou, Peking union medical college hospital, and Chongqing university cancer hospital, from December 2013 to June 2022. Pathological results were re-evaluated by ≥ 2 experienced pathologists. Patient demographics, clinical presentations, laboratory tests, and treatment regimens were systematically analyzed. This study complied with the 2013 revised Helsinki Declaration and informed consent was obtained from all the patients.

### Diagnostic criteria and clinical staging

Diagnosis was based on the 2016 World Health Organization (WHO) pathological diagnostic criteria for Hodgkin lymphoma, confirmed through morphological and immunohistochemical analysis. All patients were co-infected with HIV.

Clinical staging was performed according to the 2014 Lugano staging criteria, based on the extent of disease involvement and the presence of B symptoms. B symptoms include: (1) unexplained fever, temperature > 38 ℃ for more than 3 consecutive days, excluding infection; (2) night sweats (soaking clothes); (3) weight loss > 10% of body weight within the six months prior to diagnosis. Patients were divided into groups with or without B symptoms based on their clinical presentation.

### Treatment and response assessment

All patients received the ABVD regimen (doxorubicin, bleomycin, vinblastine, and dacarbazine): doxorubicin 25 mg/m^2^ on days 1 and 15; bleomycin 10 mg/m^2^ on days 1 and 15; vinblastine 3 mg/m^2^ on days 1 and 15; dacarbazine 375 mg/m^2^ on days 1 and 15. The median number of chemotherapy cycles was 5 (ranging from 2 to 12). All patients were administered cART. cART included two nucleoside reverse transcriptase inhibitors and one non-nucleoside reverse transcriptase inhibitor. ^18^F-fluorodexyglucose positron emission tomography/computed tomography (PET/CT) was performed for radiological evaluation. Treatment response was primarily assessed according to the 2014 Lugano criteria [5], classified as complete response (CR), partial response (PR), stable disease (SD), or disease progression (PD).

### Follow-Up

Follow-up was conducted by reviewing inpatient medical records and telephone interviews, with a follow-up cut off date of March 31, 2023, and a median follow-up duration of 46.8 months (ranging from 4.0 to 112.9 months). Progression-free survival (PFS) was defined as the time interval from HIV-HL diagnosis to disease progression, death, or the last follow-up. Overall survival (OS) was defined as the time interval from HIV-HL diagnosis to patient death or the last follow-up.

### Statistical analysis

Statistical analysis was performed using SPSS 22.0 software, and graphs were generated using Graph Pad Prism Version 9.0.0. Continuous data were presented as median (range), and categorical data were presented as frequencies (%). Survival analysis was conducted using the Kaplan-Meier method.

## Results

### Patient characteristics

Out of the 19 patients, 17 were male, with a median age of 43 years (range: 22–74 years). Pathological types included 17 cases of mixed cellularity classical HL and 2 cases of lymphocyte predominant type. Clinical staging revealed 4 cases in stage III and 15 cases in stage IV. At the time of lymphoma diagnosis, 5 patients had an Eastern Cooperative Oncology Group performance status (ECOG PS) score > 1, 8 had elevated lactate dehydrogenase levels, and 14 had an age-adjusted International Prognostic Index (aaIPI) score of 2–3. One patient was diagnosed with central nervous system involvement, 5 had bone marrow involvement, 5 had large masses (tumor longest diameter > 7.5 cm), and 16 patients presented with B symptoms. All 19 patients acquired HIV through sexual transmission, with 10 cases attributed to male-to-male transmission and 9 cases to heterosexual transmission. In 7 cases, HIV infection was discovered at the time of lymphoma diagnosis, and they commenced standard antiretroviral therapy immediately upon HIV diagnosis. Among the remaining 12 cases, the median time from HIV diagnosis to lymphoma development was 22.2 months (range, 4.3–56.4 months). These patients were already on antiretroviral therapy before the onset of lymphoma and continued treatment after lymphoma diagnosis. The median CD4 + T cell count at lymphoma diagnosis was 110 × 10^6 /L (range, 24–434 × 10^6 /L), with 5 cases having counts < 50 × 10^6 /L. The median HIV viral load was 50 copies/mL (range: 0-87202 × 10^6 /L), with 5 cases having viral loads below measurable limits. Among the patients, 12 had concomitant Epstein-Barr virus (EBV) infection, and 1 had hepatitis B virus (HBV) infection. All 22 patients received standard cART. Patients diagnosed before 2020 received a combination of two nucleoside reverse transcriptase inhibitors and one non-nucleoside reverse transcriptase inhibitor for antiretroviral therapy, while those diagnosed after 2020 were treated with bictegravir/emtricitabine/tenofovir alafenamide (Biktarvy). In terms of safety, the combination of cART and ABVD chemotherapy did not exacerbate adverse reactions. The patient characteristics are summarized in Table 1.

**Table 1:** Baseline clinical characteristics of HIV-HL patients

Characteristic	Total (*n* = 19)/N(%)
Demographics	
Male, *n*	17(89.5)
Age, years, mean (range)	43(22–74)
≤60	18(94.7)
HIV factors	
HIV-transmission category	
MSM (Men having sex with men)	10(52.6)
Heterosexuals	9(47.4)
Concomitant HIV and lymphoma diagnosis	7(36.8)
HIV viral load, copies/mL, median (range)	50 (0–87,202)
Undetectable patients	5(26.3)
CD4 count, ×10^6^ /L, median (range)	110(24–434)
<50 × 10^6^ /L	5(26.3)
(50–200)×10^6^ /L	8(42.1)
>200 × 10^6^ /L	6(31.6)
CD4/CD8, below normal	12(63.2)
Concurrent Epstein-Barr virus	12(63.2)
Concurrent hepatitis B virus	1(5.3)
ART	22(100)
No ART at diagnosis	7(36.8)
Previous ART resistance	12(63.2)
Lymphoma factors	
Lymphoma subtype	
Mixed cellularity	17(89.5)
Lymphocyte predominant	2(10.5)
ECOG PS > 1	5(26.3)
Stage 3–4	19(100)
Elevated LDH	8(42.1)
aaIPI 2–3	14(73.7)
CNS/leptomeningeal involvement	1(5.3)
Bone marrow involvement	5(26.3)
Bulky disease	5(26.3)
B symptoms	16(84.2)
Treatment factors	
Chemotherapy regimen	
ABVD	19(100)
CNS prophylaxis	1(5.3)
ART use	19(100)
Concurrent ART with chemotherapy	19(100)

### Clinical presentation

Among the 19 patients, 14 initially presented with superficial lymph node enlargement. Specifically, 10 had cervical lymph node enlargement, 2 had axillary lymph node enlargement, and 2 had inguinal lymph node enlargement. Four patients presented with fever, with 2 of them having mediastinal lymph node enlargement and 2 having retroperitoneal lymph node enlargement. One patient presented with severe anemia and bone marrow involvement.

### Treatment efficacy and outcomes

**Fig. 1:**
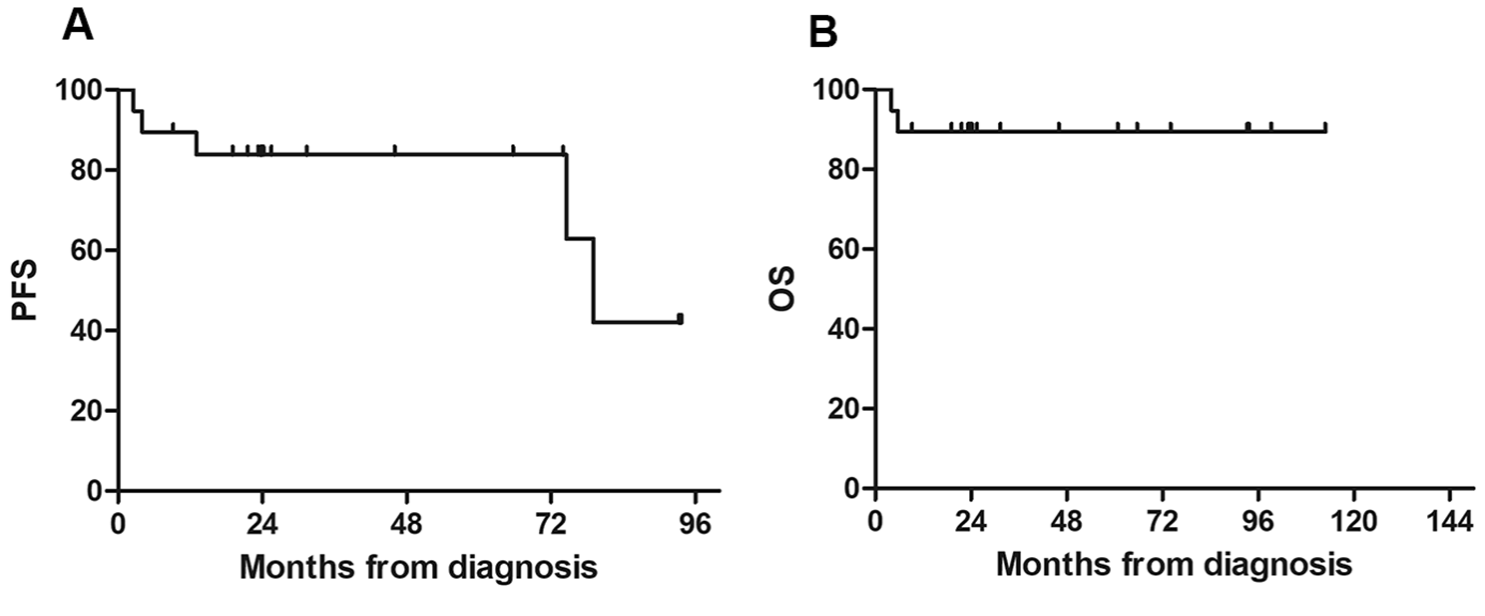
Progression free survival (A) and overall survival time (B) in HIV-HL patients

All 19 patients received cART combination with the ABVD regimen chemotherapy. Among them, 9 achieved CR, 10 achieved PR, resulting in an overall response rate (ORR) of 100%. Two patients died from severe infections during treatment, while the remaining 17 survived. The median follow-up duration was 46.8 months (range, 4.0-112.9 months) for the 19 patients, with a 5-year PFS rate of 83.9% and a 5-year OS rate of 89.5% **(**Fig. 1**).**

## Discussion

The World Health Organization categorizes tumors related to HIV infection into AIDS-defining and non-AIDS-defining tumors. In 1993, based on the cancer risk in AIDS patients, the U.S. Centers for Disease Control and Prevention classified Kaposi’s sarcoma, non-Hodgkin lymphoma, and invasive cervical cancer as AIDS-defining tumors, while cancers such as liver cancer, Hodgkin lymphoma, anal cancer, lung cancer, skin cancer, and colorectal cancer were designated as non-AIDS-defining tumors [6]. The incidence of HIV-HL has been increasing over the years, with the relative risk of Hodgkin lymphoma in HIV-infected individuals being 5–26 times higher than in the general population, with an annual incidence of approximately 50/100,000 [7–8].

HIV-related Hodgkin lymphoma is a rare subtype of lymphoma, and currently, there is a lack of large-sample retrospective clinical studies. In China, it is primarily discussed in the form of case reports, and there is no consensus on its clinical characteristics and risk factors. Louarn et al. [1] conducted a retrospective analysis of 109 cases of HIV-related Hodgkin lymphoma patients with a median age of 46 years (range, 41–51 years), where the majority were males (84%) and in stages III/IV (73%). In our study of 22 patients, the gender and age distribution is consistent with the literature, with a higher proportion of stage III-IV patients than reported in the literature.

Since the 1990s, with the widespread use of cART, the life expectancy of HIV-infected individuals has significantly increased [9–11]. A large cohort study from Denmark followed 5,701 HIV-infected individuals and 28,505 matched healthy individuals and found that the median age at death increased from 34.5 years in 1995–1996 to 73.9 years in 2010–2015 [12]. HIV-HL patients, when treated with standardized antiretroviral therapy in combination with lymphoma treatment, can achieve survival rates comparable to those of HIV-negative Hodgkin lymphoma patients [13–14]. In terms of treatment and prognosis, our study included 19 patients treated with the ABVD chemotherapy regimen, and overall, the treatment was highly effective. Among the 19 patients, 9 achieved CR, 10 achieved PR, and 17 patients survived long-term, with only 2 succumbing to severe infections. With a median follow-up of 46.8 months (range, 4.0-112.9 months), the 5-year PFS rate was 83.9%, and the 5-year OS rate was 89.5%. This suggests that the use of the ABVD chemotherapy regimen for HIV-HL results in good treatment outcomes and high survival rates.

## Conclusion

In summary, our multi-center study suggested that HIV-HL often presents in superficial lymph nodes, is characterized by high invasiveness, and exhibits favorable prognosis when treated with standardized antiretroviral therapy in combination with ABVD chemotherapy. More high-quality randomized controlled studies are needed to test our findings.

### Data availability

The raw data supporting the conclusions of this article will be made available by the authors, without undue reservation.

## Data Availability

No datasets were generated or analysed during the current study.
